# Understanding the Pathogenesis of Gestational Hypothyroidism

**DOI:** 10.3389/fendo.2021.653407

**Published:** 2021-05-25

**Authors:** Oshini Mallawa Kankanamalage, Qiongjie Zhou, Xiaotian Li

**Affiliations:** ^1^ School of Clinical Medicine, Shanghai Medical College, Fudan University, Shanghai, China; ^2^ Department of Obstetrics and Gynecology, Obstetrics and Gynecology Hospital of Fudan University, Shanghai, China; ^3^ Shanghai Key Laboratory of Female Reproductive Endocrine-Related Diseases, Obstetrics and Gynecology Hospital of Fudan University, Shanghai, China

**Keywords:** gestational hypothyroidism, subclinical hypothyroidism, pregnancy, thyroid disorders, placenta, estrogen, iodine, levothyroxine

## Abstract

Pregnancy is a complex state with many endocrinological challenges to a woman’s physiology. Gestational Hypothyroidism (GHT) is an emerging condition where insufficiency of the thyroid gland has developed during pregnancy in a previously euthyroid woman. It is different to overt hypothyroidism, where marked elevation of thyroid-stimulating hormone with corresponding reduction in free thyroxine levels, is well known to cause detrimental effects to both the mother and the baby. During the past couple of decades, it has been shown that GHT is associated with multiple adverse maternal and fetal outcomes such as miscarriage, pre-eclampsia, placental abruption, fetal loss, premature delivery, neurocognitive and neurobehavioral development. However, three randomized controlled trials and a prospective cohort study performed within the last decade, show that there is no neurodevelopmental improvement in the offspring of mothers who received levothyroxine treatment for GHT. Thus, the benefit of initiating treatment for GHT is highly debated within the clinical community as there may also be risks associated with over-treatment. In addition, regulatory mechanisms that could possibly lead to GHT during pregnancy are not well elucidated. This review aims to unravel pregnancy induced physiological challenges that could provide basis for the development of GHT. During pregnancy, there is increased renal clearance of iodine leading to low iodine state. Also, an elevated estrogen level leading to an increase in circulating thyroglobulin level and a decrease in free thyroxine level. Moreover, placenta secretes compounds such as human chorionic gonadotropin (hCG), placental growth factor (PIGF) and soluble FMS-like tyrosine kinase-1 (s-Flt1) that could affect the thyroid function. In turn, the passage of thyroid hormones and iodine to the fetus is highly regulated within the placental barrier. Together, these mechanisms are hypothesized to contribute to the development of intolerance of thyroid function leading to GHT in a vulnerable individual.

## Introduction

Thyroid dysfunction during pregnancy is a well-researched area due to the detrimental effects of either profoundly low or high circulating thyroid hormones. On the lower end of the spectrum where there is an insufficiency in circulating thyroid hormones, exist three distinct conditions, namely; overt hypothyroidism (OH), subclinical hypothyroidism (SCH), and isolated hypothyroxinemia (IH). All these three conditions can exist with or without the presence of thyroid autoimmunity marked by autoantibodies such as those against thyroid peroxidases (TPOAb +) or thyroglobulin (TgAb +). There is no doubt that overt hypothyroidism should be promptly diagnosed and treated with levothyroxine in order to prevent severe maternal and fetal consequences such as fetal loss, premature birth, neurocognitive impairment of the child (1). However, necessity for screening and treatment of SCH and IH with levothyroxine during pregnancy is still unclear among clinicians. We believe, unraveling pathophysiological changes underlying SCH and IH during pregnancy, will provide foundation for or against the necessity of treatment. This review also attempts to coincide the term ‘Gestational Hypothyroidism’ (GHT) to identify these conditions as a different entity occurring during pregnancy.

## Definitions – OH, SCH, IH and GHT

In general terms, OH is defined as elevation of circulating levels of thyroid stimulating hormone (TSH) or thyrotropin with a decrease in free thyroxine (fT4). SCH is defined as elevation of circulating TSH level with normal fT4 levels. On the other hand, IH is defined as reduction of fT4 level with normal TSH level. Among pregnant women, epidemiology studies report 0.3-0.6% prevalence of OH, 18% prevalence of SCH - depending on the TSH cut-off value (2), 1.3-23.9% prevalence of IH - depending on the fT4 cut off value (3).

American Thyroid Association in 2011 and Endocrine Society in 2012, recommended to use a fixed cut off value for TSH above 2.5 uIU/ml during first trimester and 3.0 uIU/ml during second trimester to diagnose women with SCH during pregnancy. However, where available it is highly recommended to use the local TSH range and report the findings as 97.5th percentile or above for diagnosis of SCH  (4, 5). While the cut-off value for fT4 could be 5^th^ to 2.5^th^ percentile or low from the locally available range, there has been discrepancies when measuring the circulating fT4 levels during pregnancy. Thus, total T4 or fT4 index is preferred, although many studies to date use the fT4 as a reporting value (6). In this review, we reinforce the term GHT (Gestational Hypothyroidism), for both SCH and IH identified during pregnancy in women who were previously euthyroid. It is to highlight the importance of pregnancy related occurrence of SCH and IH.

## GHT Is Associated With Adverse Maternal and Fetal Outcomes

Many observational studies have shown significant association between SCH and IH with complications during pregnancy for both the mother and the baby (7). SCH during pregnancy was associated with maternal complications such as pre-eclampsia, miscarriage, placental abruption and fetal complications such as premature delivery, neonatal death (8–10). Some other studies report that SCH is not associated with adverse neurocognitive development of the child (11, 12). Interestingly, many studies have shown that maternal IH was associated with adverse neurocognitive developmental outcomes marked by delays in mental, cognitive, psychomotor assessments and neurobehavioral outcomes including Attention Deficit Hyperactivity Disorder (ADHD), autism spectrum disorder (11–14). IH was also associated with premature delivery  (15). These differences of outcomes are pointing towards altered pathophysiology between SCH and IH during pregnancy. Upon meta-analysis evaluating negative outcomes of SCH, Taylor and colleagues (16) have suggested that SCH alters the metabolic environment and thus leads to adverse pregnancy complications while IH affects fetal neuronal development due to impaired fT4 availability. Animal studies support the latter by showing atypical neuronal migration and structural changes in brain regions such as somatosensory cortex and hippocampus among the offspring of rat mothers subjected to hypothyroxinemia as a result of low iodine intake (17). However, it is important to note that these are observational studies where correlation does not equal causation. Arguably, some studies show SCH is also associated with lower intellectual development of the infant marked by low performance of neuropsychological and behavioral tests (1, 18, 19). Of note, there are lesser number of studies that examine women with IH compared to that of SCH (16). It could be because in some countries, TSH is analyzed first and only those women with TSH aberrations are next considered for analysis, there by missing a proportion of women with IH. An evolving hypothesis - pathogenesis of gestational hypothyroidism

It is well apparent the existence of differential mechanisms for adverse outcomes observed in women with GHT. It is important to dissect these pregnancy-induced mechanisms not only to reinforce our understanding but also to explore etiology-specific alternative modes of management of GHT. Two overarching schools of hypothesis for underlying pathophysiology of GHT could be considered (20). Firstly, development of GHT directly causes the observed adverse maternal and fetal outcomes. Thus, can be managed timely with prompt administration of levothyroxine treatment. Secondly, pregnancy induced pathophysiological changes, such as mal placentation, lead to both GHT and pregnancy related complications. Thus, the observed adverse maternal and fetal outcomes are not improved with levothyroxine treatment. The latter seems more promising given the recent advances summarized in the sections below. This broad discussion is divided into two sections

- Section 01 – Regulation and feedback loops that includes the role of placenta, role of iodine and the role of estrogen
- Section 02 – Treatment of GHT with levothyroxine – an ongoing debate.

**Figure 1:**
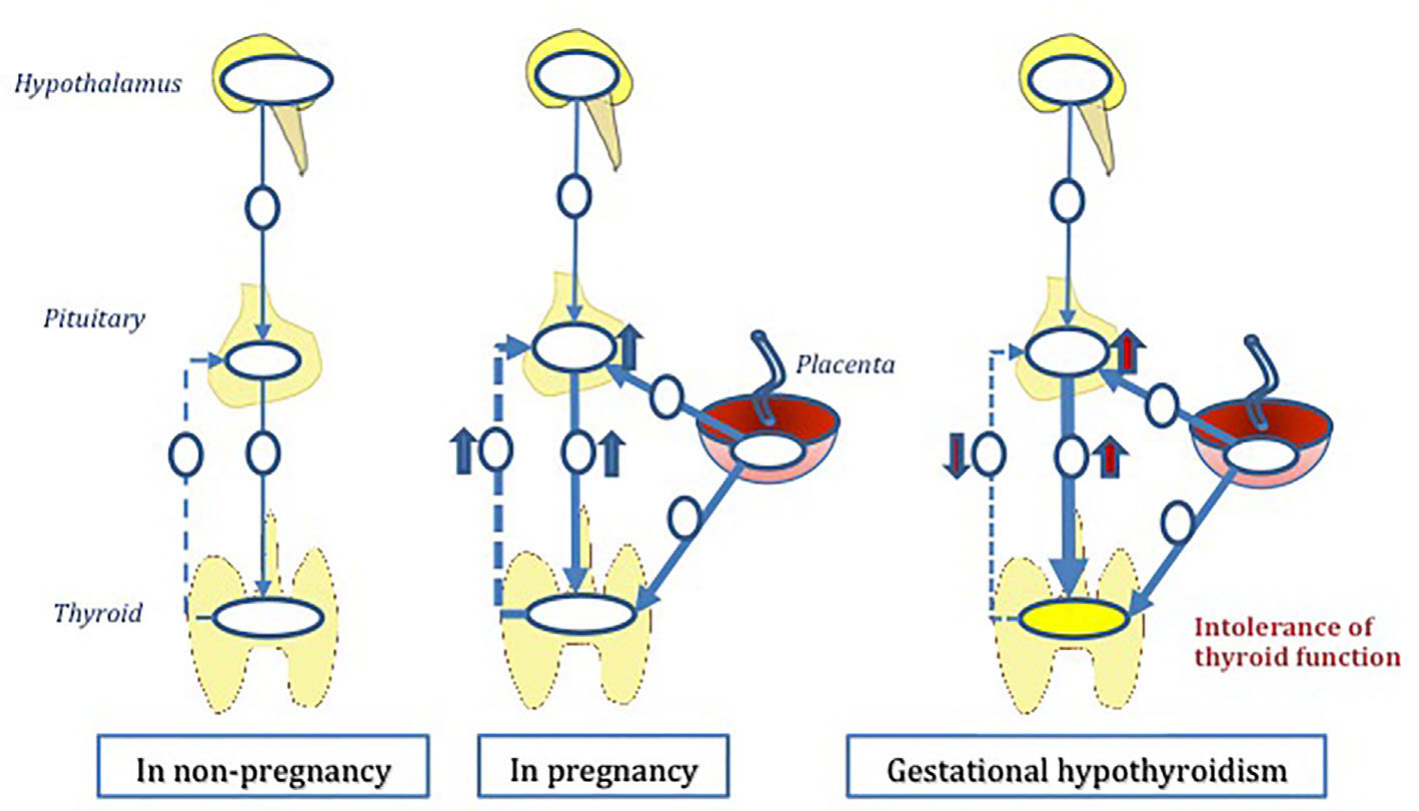
The hypothesis of ‘gestational hypothyroidism’ (GHT). GHT is developed due to thyroid regulatory mechanisms during pregnancy. Hypothalamus-pituitary-thyroid axis (HPT axis) is a deviated from its normal function as a result of multiple pregnancy induced pathophysiological insults. Mal placentation plays a central role in this multifactorial hypothesis.

The normal thyroid physiology is regulated *via* the hypothalamus-pituitary-thyroid (HPT) axis. During pregnancy demand and supply of thyroid hormone increases due to various pregnancy induced changes. We hypothesize that in some vulnerable individuals, HPT axis fail to adjust to these additional requirements - physiological or pathological, thus leading to GHT. The role of placenta in thyroid hormone regulation is of great importance in this suggested hypothesis (**Figure 1**). The sections below attempt to unravel pregnancy induced changes that could possibly impact the thyroid homeostasis, thus inducing GHT.

## Section 01 – Regulation and Feedback Loops

### Role of Placenta

#### Effect of Thyroid Hormones on the Placenta

It is important to identify that during pregnancy; placenta may play a central role in responding to and regulating maternal thyroid hormones. A recent review by Adu-Gyamfi et al. (21) well explains the available evidence on the effect of thyroid hormones on the placenta. Of interest, in established conditions which are due to abnormal placentation, such as pre-eclampsia, miscarriage, IUGR; studies have found that these women show abnormal levels of thyroid hormone as well. It is understood that optimal level of thyroid hormone is necessary for proliferation and differentiation of cytotrophoblasts (21). Furthermore, thyroid hormone might also regulate extravillous trophoblast invasion as shown by failure of extravillous trophoblasts to migrate and invade the decidua in pregnancies of hypothyroid rats (22). mRNA expression of molecules such as metalloproteinases (MMP2 and MMP3), oncofetal fibronectin and integrin alpha5beta1, which contribute to invasiveness of extravillous trophoblasts, have found to be increased in extravillous trophoblasts in-vitro when treated with T3. In maternal hypothyroid state, there is also alteration to the anti-inflammatory environment within the placenta, marked by reduced expression of IL-4, IL10 in the decidua  (21). If any of the above processes are dysregulated, due to alternation to thyroid hormone availability, it may lead to a dysfunctional placenta.

#### Regulation of Thyroid Hormones Within the Placenta

Availability of the thyroid hormone within the placenta is governed by regulation of iodine, thyroid hormone metabolism and transport. A recent study by Peng et al. (23), examines the divergence of iodine and thyroid hormones in a term placenta. Placenta is the only other organ, other than the thyroid gland, that stores iodine. It is believed this strict regulation of iodine transport is to ensure that fetus is protected from excess exposure to iodine. However, fetus does require iodine to produce its own thyroid hormones. Iodine is obtained *via* uptake of maternal iodine, placental deiodination of thyroid hormones and the capacity of the placenta to store and transport iodine to fetal circulation. It is believed that there is a similar mechanism to thyroid gland by which placenta transport iodine. Influx of iodine is thought to be governed by Sodium/Iodine symporter (NIS) while efflux is governed by pendrin. In their study by Peng et al. (23), NIS was highly expressed in the apical membranes of syncytriotrophoblasts, the fetal side of the placenta while pendrin was detected syncytriotrophoblasts as well as cytotrophoblasts layers of placenta.

Deiodinases are set of enzymes that metabolize thyroid hormones. Deiodinases type 2 and 3 are found in the placenta, while D3 is predominant. D3 converts T4 to rT3 by inner ring deiodination of T4 and converts T3 to T2, there by releasing iodine (21). D3 is highly expressed in the syncytiotrophoblasts and cytotrophoblast layers of term placenta, fetal endothelium and decidua which in contact with the maternal circulation. This optimal position is to ensure that fetus obtains adequate iodine supply and release to fetal circulation (23). During the first trimester, differential expression was observed where D2 is strongly expressed in the cytotrophoblasts layer and weakly in the syncytiotrophoblast layer. On the other hand, D3 is weakly expressed in the cytotrophoblast layer and strongly in the syncytiotrophoblast layer (21). Given that D3 is responsible for inactivation of thyroid hormone, it can be argued that over expression of D3 lead to a reduction in the placental thyroid hormone availability.

With regards to the placental regulation of thyroid hormone transport, thyroid hormone must pass through apical layer, basal layer of syncytiotrophoblasts, pass intracellularly through cytotrophoblasts and reach the fetal endothelium. On the maternal side, high levels of T3 and T4 than the fetal side of placenta are found. Minimal amounts of T3 and T4 is transported from maternal to fetal side. On the fetal side of the placenta, rT3 levels are increased, which plays a main role in ensuring low levels of T3 (23). Considering all together, it shows that thyroid hormone availability to the fetus is tightly regulated by the placenta. Six thyroid hormone transporters are known to exist within the placenta; namely, large amino acid transporter -1 (LAT1), Large amino acid transporter - 2 (LAT2), Organic anion transporting polypeptide 1A2 (OATP1A2), Organic anion transporting polypeptide 4A1 (OATP4A1), Monocarborxylate transporter - 8 (MCT8), Monocarboxylate transporter - 10 (MCT10). Differential expression of these transporters between maternal and fetal side of the placenta is found and abnormal expression of these transporters are thought to contribute to abnormal placentation (21).

#### Response of Maternal Thyroid Function to Placental Derived Factors

The above mentioned are the placental response to thyroid hormone levels. Strong evidence exists to show dysfunctional placentation due to overt hypothyroidism and hyperthyroidism in pregnant women (21). The question remains whether any factors secreted by the placenta can alter the maternal thyroid function, perhaps lead to gestational hypothyroidism. Presence of GHT in turn could lead to associated maternal and fetal complications. Indeed, a promising set of studies within the last few years, attempt to clarify this notion. Thus far, placental human chorionic gonadotropin (hCG), Placental growth factor (PIGF), pro-angiogenic factor and soluble FMS-like tyrosine kinase-1 (s-Flt1 or soluble vascular endothelial growth factor receptor-1), an antagonist of VEGF and PIGF signaling are identified as potential regulators of maternal thyroid function.

##### Human Chorionic Gonadotropin (hCG)

Placenta produces hCG which shares molecular similarity with TSH. This homology between the beta subunit of hCG and TSH allows for stimulation of the thyroid gland by binding and activating the TSH receptors of thyroid follicular cells and exerting its effects *via* intracellular messengers, such as cAMP (9). It is believed that both the amplitude and duration of hCG peak plays a role in the degree of the stimulation of the thyroid gland. A transient suppression of TSH and elevation of serum fT4 is expected in those with high concentration of hCG lasting for a longer duration. This may be evident from studies that suggest hCG has a causative role in hyperemesis gravidarum, where excessive vomiting and associated 5% weight loss is considered to be due to transient mild gestational hyperthyroidism state (24). Korevaar and colleagues (25) also support this notion by demonstrating that serum hCG concentration is associated significantly with increasing risk of SCH and OH. As discussed previously, the requirement for thyroid hormone production markedly increases during early stages of the pregnancy, thus hCG plays a major role in supplying for this increased demand (6). Question thus arises whether the sensitivity of TSH reduces at the thyroid gland and whether those vulnerable individuals fail to adapt at a pituitary level. Of interest, a recent analysis of population based prospective cohort study of more than 5000 pregnant women, also mentioned above, demonstrates that serum hCG concentration is not associated with risk of SCH during pregnancy (measured by TSH levels) but it is associated with lower risk of IH (25). However, those women with SCH is presumed to have a lack of thyroidal capacity to respond to hCG stimulation, as shown by an attenuated response to hCG mediated increased fT4 concentrations compared to euthyroid women. Although, there are no clinical trials showing a direct impact due to obvious ethical reasons, it can be speculated that this impaired rise of fT4 in response to increasing hCG, could further dysregulate TSH secretion by the pituitary gland, potentially worsening the SCH state in these women. Interestingly, external factors such as higher BMI, male fetal sex, and parity >2, were associated with reduced thyroidal response to increase in hCG concentration.

##### Placental Growth Factor (PIGF) and Soluble FMS-Like Tyrosine Kinase-1 (s-Flt-1)

PIGF is a pro-angiogenic factor produced by the placenta which shares 53% molecular homology with vascular endothelial growth factor (VEGF) while s-Flt1 is an anti-angiogenic factor produced by the placenta that can antagonise the effects of PIGF and VEGF. These two factors are potent regulators of vascular endothelial homeostasis within the placenta. Dysregulation of these factors, such as high sFLT1 to low PIGF ratio, has been implicated in pregnancy complications such as pre-eclampsia, IUGR and small for gestational age (26, 27). Thyroid gland is a highly vascularized organ, where animal studies have shown that there is a significant reduction in thyroid vasculature in the presence of VEGF blockade (28). More interestingly, in those patients who underwent VEGF inhibition as anti-angiogenic cancer therapy, have shown to develop OH or SCH as a side effect (29). Firstly, Korevaar and colleagues (30) determined the effects of placental derived factors PIGF and sFlt1 on newborn thyroid function. They found that while elevation of PIGF was associated with fT4 positively, increasing levels of sFlt1 was associated with decreasing levels of fT4 and increasing levels of TSH in newborn cord blood samples. Upon significant association with newborn thyroid function, Korevaar and colleagues (31) then determined the effects on maternal thyroid function. Interestingly, while they indeed found sFlt1 to show a significant negative linear association with fT4 levels, there was a non-significant positive linear association with TSH level. However, very high levels of sFlt1 were associated with a 2.4 fold increased risk of SCH, suggesting a threshold effect of sFlt1 on TSH levels. Supportive evidence by a nested case control and population-based study in pre-eclamptic women by Levine et al. (32) shows that maternal higher sFlt1 level was significantly associated with larger increases in TSH levels between early pregnancy and pre-delivery, suggesting SCH in women with pre-eclampsia. PIGF, on the other hand, was associated with a negative trend in both TSH levels and fT4 levels (31). This is different to the observed effect on the fetal thyroid gland. Authors speculate a differential stimulation of angiogenesis by PIGF between a fully formed maternal thyroid and newly forming fetal thyroid gland. In addition, when stratified according to TPOAb positive pregnant women, effects of sFlt1 and PIGF on TSH levels were found to be stronger, supporting a multi-risk factor model on maternal thyroid function. Furthermore, supporting this model, Korevaar and colleagues (31) analyzed the effect of sFlt1 and PIGF alongside high and low levels of hCG levels. They found that anti-angiogenic effects of high sFlt1 levels could be rescued by increasing concentrations of hCG, which is also known to be a stimulator of angiogenesis. Of interest, they found that overall effect of increasing levels of PIGF and hCG leading to a decrease in thyroid function, possibly due to a hyper stimulatory effect through a similar mechanism of action *via* stimulation of the cAMP/PKA pathway**.**

### Role of Iodine

Iodine is an essential micronutrient for the synthesis of thyroid hormone. Pregnancy, a state of many physiological changes, increases the demand for iodine around 50% (33). This is due to increased renal clearance of iodine as the glomerular filtration rate increases, relative increase in production of maternal thyroid hormone and increased fetal requirements for iodine during second and third trimester for production of its own thyroid hormones (9). There is an active transport of iodine to the thyroid gland which is regulated by TSH and by the concentration of iodine in blood. Thus, an adequate supply of dietary iodine is essential to supply for this increased demand. According to guidelines by WHO/UNICEF/ICCIDD the recommended iodine intake for pregnant and lactating women was increased by 50ug/L from normal adult range of 100-199g/L.

#### Iodine Deficiency During Pregnancy

A recent systematic review by Candido et al. (34) clearly classify the adverse effects of light, moderate and severe iodine deficiency amongst pregnant women between the three trimesters. In pregnant women with moderate iodine deficiency, subclinical hypothyroidism, pre-eclampsia, anemia, fetal growth restrictions, congenital anomalies were observed while in pregnant women with severe iodine deficiency, eclampsia, placenta previa, cretinism, miscarriage, hemorrhage and fetal demise were observed. The review found that some authors suggest during light iodine deficiency there is an increased iodine uptake to increase thyroxine secretion, noted by the hyperplasia by the thyroid gland, thus reducing the damages. However, other studies suggest even during light iodine deficiency there is an increase in circulating TSH, which is associated with increased oxidative stress probably through antagonizing effects of insulin and thus inducing hyperglycemia (35, 36). Iodine intake is grouped according to WHO criteria by taking measurements of median urinary iodine concertation (UIC). This is because more than 90% of dietary iodine intake is excreted *via* urine. These groups of iodine intake are as follows; adequate intake (UIC 150-249 μg/L), mild/light deficiency (UIC, 100-150 μg/L), moderate and severe deficiency (UIC, <100 μg/L), and more than adequate and excessive intake (UIC, ≥250 μg/L).

Association of SCH with severe and moderate iodine deficiency is evident with studies conducted over many different regions (37–40). The emerging idea is that due to iodine deficiency (UIC < 100g/L) there is an accumulation of oxidative stress that leads to an inflammatory response that in turn triggers thyroid autoimmunity. A recent study by Sun and his colleagues (41), have found an association of thyroid autoantibodies such as TPOAb and TgAb with iodine deficiency. In addition, this cross-sectional study in an iodine adequate region in China of over 7000 pregnant women has shown that in those pregnant women with isolated positivity for TgAb had higher risk of OH and SCH. In addition, as the titers of these antibodies TPOAb and TgAb increased, upward trend of TSH level and downward trend of fT4 levels were clearly observed. Serum thyroglobulin, which is an important component of thyroid hormone synthesis, was also found to be reduced in those antibody positive pregnant women, while associated with increased TSH level and decreased fT4 levels. The study suggests that serum thyrogobulin could possibly be considered as biomarker to assess the extent of thyroid damage in pregnant women. Considering it all, we can postulate that due to the increased demand for iodine and presence of masked yet sustained thyroid autoimmunity, more pronounced adverse effects are observed during pregnancy thus causing SCH or OH.

On the other hand, some studies conducted in other iodine-adequate regions, have not found a significant association between UIC and thyroid function in pregnant women (42, 43). Due to discrepancies of measurements of UIC and thyroid function between laboratories, specifically during pregnancy, outcome of all these studies should be considered with care.

#### Excess Iodine Intake During Pregnancy

At the present time, with introduction of universal salt iodization, many regions of the world have adequate supply of dietary iodine. Consensus however is that there is a chronic excess intake of dietary iodine. Interestingly, a systematic review and meta-analysis including observational studies by (44), shows that excess iodine intake is significantly associated with SCH in general population. Transient Wolff-Chaikoff effect describes how thyroid hormones level would decrease under high serum iodide concentration and return to normal level after a few days. However, it is believed that in vulnerable individuals, an escape of this adaption could occur, thus resulting in iodine-induced thyroid dysfunction with or without autoimmune thyroiditis eventually leading to SCH (44, 45). A study by Su et al. (46) have identified a group of such vulnerable reproductive-age women, defined by gene polymorphism of PDE4D in Shanxi province, China. Several different studies support the notion that dietary iodine excess during pregnancy leads to subsequent SCH (33, 35, 45, 47, 48). Due to high risk of SCH, Shi and his colleagues (48) suggests, upper limit of iodine intake during early pregnancy in iodine-sufficient regions should not exceed UIC 250g/L while a UIC of 500g/L should not be exceeded due to significantly high risk of IH. Of interest, studies supporting both mild iodine deficiency and excess iodine intake leading to SCH during pregnancy have found pre-conception higher body mass index (BMI) to be a risk factor (40, 47).

### Role of Estrogen

Changes to circulating estrogen during pregnancy could be another factor that might be contributing to development of GHT. It is known that there is relative increase in basal level of thyrogobulin in response to elevated estrogen levels. This decreases availability of free thyroid hormones, which in turn stimulates pituitary-thyroid axis. Thus, the thyroid gland is challenged to secrete more thyroid hormones to ensure a normal availability of free thyroid hormone. A study by De Geyter et al. (49) shows that women with SCH and on a fixed daily low dose (50 ug) supplementation with levothyroxine, still showed an increase in the TSH levels, reaching statistical significance compared to euthyroid women between gestational week 6-8. This closely parallels the pattern of rising estrogen levels during early pregnancy.

A retrospective cohort study at a fertility clinic by Hammond et al. (50) reports that 24% of women who were previously found to be euthyroid, developed mild hypothyroidism within 6 weeks of gestation. A strong association was found between gonadotropin stimulation in the conception cycle and mean elevated TSH level and mean low free T4 level during early pregnancy compared to those women who did not receive gonadotropin treatment. This is also supported by Benaglia et al. (51) where an increase in serum TSH level is found after ovulation trigger, in previously euthyroid women receiving controlled ovarian hyper stimulation. It is suspected that exogenous gonadotropin treatment and associated higher than normal E2 level in the normal cycle, may lead to a further increase in thyroglobulin level. Thus it could be assumed to lead to a fall in the availability of free circulating thyroid hormone (52). However, further studies are required to comment on the equilibrium state of T4 in case of increased thyroglobulin levels.

On the contrary, in a review by Mintziori et al. (53) that outlines the thyroid function and IVF outcome, it notes current in-vivo experimental evidence where 17[beta]-estradiol is shown to directly down regulate paraventricular-TRH mRNA concentration. On the other hand, higher levels of estrogen has shown to suppress T-helper cell responses type 1 (Th1), which is thought to be important for developing thyroid autoimmunity marked by increased serum concentrations of anti-thyroid peroxidase (anti-TPO) and antithyroglobulin (anti-TG) antibodies (54). Presence of thyroid autoimmune antibodies prior to conception can be considered a risk factor for GHT. Considering it all, in euthyroid women undergoing in-vitro fertilization, exist a hyperestrogenism state. For some, it may lead to an increase in serum TSH thus risking the development of GHT while others may not be vulnerable.

## Section 02 – Treatment of GHT With Levothyroxine - An Ongoing Debate

### Evidence From Studies Thus Far

#### Evidence From Randomized Controlled Trials (RCTs)

Currently there are three different randomized controlled trials that have assessed whether the treatment with levothyroxine was beneficial or not for women with GHT (55–57). When Nazapour et al. (58) stratified their cohort for thyroid antibody positivity, significant reduction in rate of preterm delivery and neonatal admission rate was noted in women with TPOAb (+) and a TSH cut off above 4.0uIU/ml. In the following year, Nazapour et al. (57) assessed their cohort based on two different TSH cut off values, 2.5mIU/ml and 4.0uIU/ml. They noted that in TPOAb (-) women with cut off value of TSH>2.5uIU/ml did not show lower rate of adverse pregnancy outcomes. However, rate of preterm delivery was reduced in TPOAb (-) women with TSH> 4.0uIU/ml who received treatment. Meta-analysis and a systemic review of these RCT trials by Yamamoto et al. (20) states that there was no significant difference between treatment with levothyroxine vs control (either placebo or no treatment) for the adverse outcomes assessed; which are preterm delivery, placental abruption, gestational age at delivery, NICU admission and infant head circumference.

#### Evidence From Combined Analysis of Cohort Studies and RCTs

On the contrary, systematic review and meta-analysis of 13 cohort studies and RCTs with total of 11,503 participants, noted that levothyroxine treatment reduced the odds of pregnancy loss and increased the chances of live birth rates in women with SCH (59). This is supported by an early systematic review and meta-analysis which found that with levothyroxine treatment for SCH, there is a significant decrease in pregnancy loss and increase in chance of live birth rates, among women who underwent assisted reproductive technologies to facilitate their pregnancy (60). However, as Nazapour et al. (59) suggest, results of their meta-analysis are to be interpreted with caution, as there are not enough RCTs. Also, there is not enough power to exclude the effect of thyroid autoimmunity among the participants (59). Similar to the meta-analysis of only RCTs by Yamamoto et al. (20), meta-analysis by Nazapour et al. (59) which also included cohort studies, did not find significance with chances of adverse maternal complications, obstetrical hemorrhage, neonatal ICU admission among women who got treated for SCH.

#### Evidence on Effect of Treatment on Neurological Development

Interestingly, all these RCT trials investigated the effect of levothyroxine treatment on neurocognitive and neurobehavioral development of the child born to mothers diagnosed with GHT (55–58). It is of importance to note that these trials did not find benefit of treatment on neurological development of the child at 3, 5 and 9 years of age. In addition, no benefit of time of initiation of therapy (prior to 14 weeks and prior to 11 weeks) on childhood IQ was noted (20). This was further supported by a recent prospective cohort study where preconception treatment with levothyroxine shown to be not associated with improved neurocognitive function in children up to 2 years of age (61). Recently, CATS-I study by Lazarus et al. (56), the first RCT trial to be performed, was re-assessed for ADHD and ASD in children of mothers who were treated and not treated for GHT (62). No significant difference was seen even when stratified for SCH and IH separately. These studies are summarized in **Table 1** and **Table 2**.

**Table 1:** Summary (part 1) of the studies that have assessed neurocognitive and neurodevelopment of the child born to mothers with Gestational Hypothyroidism (GHT) and treated with appropriate dose of levothyroxine throughout pregnancy.

Type	Name of the study	Definition of GHT	Sample size	Treatment	Time of initiation of therapy
RCT*	Lazarus et al. (56) CATS-I trial	TSH* > 97.5th percentile Or/and fT4* < 2.5th percentile	794	Starting dose of 150ug of levothyroxine per day (adjusted as necessary)	Median gestational age of 13 weeks + 3days
RCT	Hales et al. (63) CATS-II trial	TSH > 97.5th percentile Or/and fT4 < 2.5th percentile	449	Starting dose of 150ug of levothyroxine per day (adjusted as necessary)	Median gestational age of 13 weeks + 3days
RCT	Hales et al. (62) (CATS-I trial cohort)	TSH > 97.5th percentile Or/and fT4 < 2.5th percentile	475	Starting dose of 150ug of levothyroxine per day (adjusted as necessary)	Median gestational age of 13 weeks + 3days
RCT	Casey et al. (55)	SCH* –> TSH >= 4.0mU/L with normal fT4	677	Starting dose of 100ug of levothyroxine (adjusted as necessary)	Mean gestational age of 16.7 weeks
		IH*–> fT4 < 0.86ng/dL with normal TSH	526	Starting dose of 50ug of levothyroxine (adjusted as necessary)	Mean gestational age of 17.8 weeks
Prospective Cohort	Zhou et al. (61)	TSH > 97.5th percentile and TPOAb (+) *	466	Levothyroxine treatment targeting TSH values of 0.1-2.5mU/L	Before conception and after conception (mean gestational age 13.5 weeks)

*RCT, Randomised Controlled Trial; TSH, Thyroid-Stimulating Hormone; fT4, Free Thyroxine; SCH, Subclinical Hypothyroidism; IH, Isolated Hypothyroxinemia; TPOAb (+), Thyroid Peroxidases antibodies.

**Table 2:** Summary (part 2) of the studies that have assessed neurocognitive and neurodevelopment of the child born to mothers with Gestational Hypothyroidism (GHT) and treated with appropriate dose of levothyroxine throughout pregnancy.

Name of the study	Child neurological outcome measured	Assessments used	Result comparing intervention vs control/placebo groups
Lazarus et al. (56) ‘CATS-I trial’	IQ at age 3	Wechsler preschool and primary scale of intelligence (2003 edition) Child behavior aspects that may affect IQ is measured by Child Behaviors Checklist (CBCL 2000) Achenbach System of empirically based assessment, University of Vermont Behaviors Rating Inventory of Executive Function, preschool version, 2003 Child Behavior Checklist at 36months and 60 months of age - behavior and social competency assessment	No significant difference of the IQ scores of children
Hales et al. (63) CATS-II trial	Full Scale IQ at age group 7-10 years old	Wechsler Intelligence Scale for Children, Fourth Edition UK (WISC-IV) calculated equally from verbal, perceptual reasoning, working memory IQ and processing speed IQ domains.	No significant difference in IQ scores of children
	Long term memory, working memory, fine motor coordination	Developmental Neuropsychological Assessment (NEPSY), Second Edition		
Hales et al. (62) (CATS-I trial cohort)	ADHD features in children 7-10 years of age (emotions, conduct, hyperactivity, peer problems, prosocial, inattention, impulsivity, social communication)	The Strengths and Difficulties Questionnaire (SDQ) The Child ADHD Questionnaire (ADHDq) Social Communication Questionnaire (SCQ) All 3 questionnaires were completed by the mothers of the children	No significant differences of ADHD features between the children
Casey et al. (55)	Full Scale IQ at 5 years of age Secondary Outcomes - Cognitive, motor, language, behavior and social scores	Wechsler Preschool and Primary Scale of Intelligence III (WPPSI-III) Differential Ability Scales - II (DAS) at 3 years of age if the WPPSI-III score was not available at 5years. Bayley Scales of Infant Development, Third Edition (Bayley - III) at 12 months, 24months Conner’s Rating Scales - revised at 48months of age for assessment of attention	No significant differences seen with cognitive and behavioral outcomes
Zhou et al. (61)	Psychological Development assessment at age 6, 12, 24 months	Chinese Version of Gesell development Diagnosis Scale (GDDS) with 4 domains - motor, adaptability, language and social emotional responses	No significant difference of neurocognitive development of children between different time of initiation of therapy (before conception and after conception)

### Choice of Treatment of GHT?

#### Recommendations From Endocrine Society and American Thyroid Association

Therefore, the choice of treatment of GHT with levothyroxine is becoming increasingly controversial. It was long believed that levothyroxine therapy is within physiological range; as such, there is no harm if chosen to treat women presenting with SCH during pregnancy. Thus, it has been the recommendation from the Endocrine Society to treat all women with SCH irrespective of the presence of thyroid autoantibodies (5). Of note, RCT by Casey et al. (55) does not report negative outcomes amongst the women treated with levothyroxine. However, this contrasts with the recommendation from American Thyroid Association (ATA) which recommends treatment only for SCH with TPOAb (+) and not for IH (4).

#### Risk of Over-Treatment With Levothyroxine

In the recent past, studies have emerged challenging the treatment with levothyroxine for SCH during pregnancy. Over-treatment with levothyroxine that lead to an increase in fT4 levels, though at a subclinical level, is associated with increased risk of pre-eclampsia (64). It is further supported by an USA national survey that demonstrates while there is a reduced risk of miscarriage, other complications such as preterm delivery, gestational diabetes and pre-eclampsia show an increased risk among the women who were treated with levothyroxine for SCH (65). In addition, increased concentration of fT4 has been found in fetal blood during cordocentesis (14). Of note, paradoxically, it is shown that such supra-normal levels are associated with low IQ, low grey matter and cortex volume in the child (66). Supporting this notion, the re-assessment of CATS-I RCT trial, showed that over treatment with levothyroxine, defined as an increase in fT4 levels above the 97.5th percentile, led to more conduct, ADHD features and ASD in children of mothers treated (62). Furthermore, in support, a cohort study in Denmark demonstrated that the children of mothers with subclinical hyperthyroidism throughout their pregnancy had increased the risk of being diagnosed with ADHD (67).

#### Monitor TSH and fT4 Upon Initiation of Treatment

This apparent ‘bi-phasic’ effect of fT4 both low and high levels, though subclinical, causing adverse outcomes, stresses the importance of careful monitoring of TSH as well as fT4 levels if chosen to treat women with GHT. It is further facilitated by these RCT studies that shows no benefit of treatment of GHT, questioning the reasoning for initiation of treatment (20). In the light of emerging findings, a recent review by Taylor et al. (16) attempts to introduce a stepwise algorithm yet specific, when initiating the treatment for either SCH or IH during gestation. Importantly they suggest monitoring TSH and fT4 levels every 4 weeks to maintain optimal circulating concentrations of fT4 (16).

## Discussion

GHT is a separate entity of thyroid hormone dysregulation occurring during pregnancy. Aberrations of iodine regulation, elevated estrogen levels, mal placentation or physiological placental derived factors insult the maternal thyroid hormone regulation and function, possibly leading to GHT. Current research studies do not fully elucidate the pathological mechanisms behind the development of GHT. It is evident that more RCT trials, more *in vitro* and *in vivo* studies are required to unravel the complex hormonal dysregulations and their downstream effects leading to GHT. This review identifies the following research gaps in the field of GHT.

- Although adverse maternal and fetal effects of OH are well described, complications occurring with the existence of SCH and IH are not fully understood. In fact, differential modes of analysis of neurodevelopment of the offspring between studies, especially the RCTs, creates discrepancies and questions the outcome of the studies. Thus, a consensus has to be reached with the mode of analysis. Also presenting results with combined techniques such as MRI and genetic testing could be considered.
- For other endocrine abnormalities during pregnancy such as gestational diabetes, glucose control and treatment is well outlined. However, the aim of treatment of GHT with levothyroxine remains controversial. There appears a fine balance of over treatment and under treatment. Furthermore, time of initiation of screening and therapy remains to be understood.
- It is also important to consider whether to continue treatment postpartum, if levothyroxine treatment is initiated for GHT. Prospective cohort studies on continuation of treatment postpartum could shed light on thyroid function and other related complications of those women on therapy.
- In addition, there is inadequate advice on timeline of follow up of these women identified with SCH and IH during their pregnancy. Other pregnancy related endocrine abnormalities such as gestational diabetes is strictly followed up with glucose level many years post pregnancy. However, there is a lack of research studies following up the maternal thyroid function, endocrine and metabolic abnormalities of women with GHT later in their life.

## Author Contributions

XL -Proposing the idea of gestational hypothyroidism and identifying discrepancies among treatment options for women, highlighting the need for review, final editing of the article. QZ - Outline of the article, setting rationale, continuous guidance, identifying gaps in research, discussion, figures, final editing of the article. OM - Gathering primary research articles, analyzing and writing up the review. All authors contributed to the article and approved the submitted version.

## Funding

This study was supported by the Shanghai Key Program of Clinical Science and Technology Innovation (No. 17411950500, No. 17411950501 and No. 18511105602), Clinical Research Plan of SHDC (SHDC2020CR1047B and SHDC2020CR6021), the Shanghai Excellent Young Scholar Plan of Public Health (2020-2022, GWV-10.2-YQ13), Elite Young Scholar 2025 of Fudan University (2020-2023), National Science Foundation of China (81741047), Shanghai Medical Center of Key Programs for Female Reproductive Diseases (2017ZZ01016). The funders had no role in study design, data collection and analysis, decision to publish, or preparation of the manuscript. Database reported in this publication was supported by Chinese government, a national nonprofit project which benefit rural reproductive aged population.

## Conflict of Interest

The authors declare that the research was conducted in the absence of any commercial or financial relationships that could be construed as a potential conflict of interest.
